# Myocardial Fibrosis: Assessment, Quantification, Prognostic Signification, and Anti-Fibrosis Targets: A State-of-the-Art Review

**DOI:** 10.3390/jcdd12050192

**Published:** 2025-05-18

**Authors:** Salvatore Poddi, Cynthia L. Lefter, Daniele Linardi, Andrea Ardigò, Giovanni B. Luciani, Alessio Rungatscher

**Affiliations:** 1 Division of Cardiac Surgery, University of Verona Medical Center, 37126 Verona, Italy; salvatore.poddi@univr.it (S.P.); giovanni.luciani@univr.it (G.B.L.); 2 George Emil Palade University Medical School, 540139 Targu-Mures, Romania; cynthia_lefter@yahoo.com; 3 Division of Cardiothoracic Surgery, Mater Misericordiae University Hospital, D07 R2WY Dublin, Ireland

**Keywords:** myocardial fibrosis, assessment, quantification, heart failure, predictive value

## Abstract

Myocardial fibrosis (MF) is the excessive deposition of extracellular matrix (ECM) from myofibroblasts and is crucial in patients with heart failure (HF). Much work is still needed to fully understand its features and clinical role. This review aims to summarize the state-of-the-art of MF knowledge, focusing on assessment, quantification, predictive value, and future therapies. We performed a literature search about MF studies published between 2014 and 2024, including clinical studies on MF assessment or quantification, the role of MF as a prognostic factor in patients with HF, basic science studies on fibrosis assessment, and the role of the main mechanisms involved in MF. We identified 5161 potentially relevant articles. After excluding non-appropriate ones, we had 186 potentially suitable studies. After full reading and a review of references, 40 articles were included in our review: 8 were about MF assessment, 5 about quantification, and 27 about fibrosis as a prognostic factor. MF is a crucial process in patients with cardiac diseases and leads to HF and arrhythmias. Assessment and quantification have taken great steps forward, but more research is needed to strengthen MF’s role as a prognostic factor in the future. Basic science will play a key role in anti-fibrosis treatment.

## 1. Introduction

Myocardial fibrosis (MF) is a complex process which develops secondary to chronic inflammation and/or mechanical stress. It is a compensatory response that later leads to histological changes that cause cardiac dysfunction with or without reduced left ventricular ejection fraction (LVEF) [1]. MF is defined as the excessive deposition of extracellular matrix (ECM) from active myofibroblasts [2]. In terms of pathophysiology, we may describe a replacement MF, interstitial MF, and perivascular MF. Replacement fibrosis is reparative and reflects the absence of regenerative capacity of the adult mammalian heart in response to primary necrotic injury. Interstitial and perivascular fibrosis may result from prolonged activation of fibrogenic stimuli and may represent primary injurious processes. Replacement fibrosis is predominantly associated with systolic dysfunction; interstitial and perivascular fibrosis typically reduce left ventricular compliance and then alter diastolic function, with less pronounced or late effects on systolic function [3]. Interstitial and perivascular fibrosis are sequential lesions which are sometimes called reactive fibrosis; they generally occur in pathological conditions such as pressure or volume overload and diabetes mellitus. We may further simplify by stating that replacement MF develops after acute myocardial infarction as a scar to replace infarcted myocardium; reactive MF develops after volume and/or pressure overload, as a compensatory mechanism [3,4].

Cardiac fibroblasts are the main effectors of heart response to injuries, involved in ECM production and degradation, inflammatory cell recruitment, and scar formation [5]. Activated fibroblasts (myofibroblasts) are the crucial cellular effectors of MF, serving as the main ECM producers [6]. Predominant components of the adult human heart ECM are collagen type I (85%) and collagen type III (11%); excess deposition of ECM proteins reduces ventricular compliance and worsens HF. Moreover, excess ECM deposition and fibroblast proliferation alter the mechano-electric coupling of cardiomyocytes, increasing the risk of arrhythmia and mortality [7].

MF is then a crucial process in patients with heart disease. However, despite important steps forward over the last years, much work is needed to fully understand its features and clinical role. In this review, we summarize the state-of-the-art of MF knowledge, focusing on fibrosis assessment, quantification, and its significance as a negative prognostic factor, mostly in patients with cardiovascular diseases. Moreover, we discuss the potential role of fibrosis as a therapeutic target.

## 2. Methods

### 2.1. Study Design

This is a review including both observational clinical and basic studies about the assessment and/or quantification of myocardial fibrosis and its role as a prognostic factor. The present study has been conducted following the current PRISMA protocol and guidelines for systematic reviews and meta-analysis.

### 2.2. Search Strategy

We performed a literature search using one database (PubMed) to find studies about MF which were published between 2014 and 15 September 2024. The following keywords were used for the detailed research: “myocardial fibrosis assessment”; “myocardial fibrosis quantification”; “myocardial fibrosis significance”; “myocardial fibrosis prognostic”; “myocardial fibrosis” AND “predictive value” AND/OR “complications”; “myocardial fibrosis” AND “cardiovascular events”; “myocardial fibrosis” AND “prognosis” AND/OR “outcome”; and “cardiac MRI” AND “fibrosis quantification”. Only full articles available in the English language were considered for the full text review.

### 2.3. Selection Criteria

We included observational, clinical studies (both prospective and retrospective), clinical trials, reviews, and meta-analysis about MF assessment and/or quantification and about the role of MF as a prognostic factor for poor outcomes (specifically death, malignant arrhythmias, major complications) in patients with HF. We also included basic science studies (laboratory studies, in vitro and in vivo) about both fibrosis assessment and the role of the main mechanisms involved in MF. We excluded studies which were case reports, editorials, letters to editors, and studies that did not meet our previously described inclusion criteria. The eligibility of studies was independently analyzed by three reviewers (SP, CL, AR), first by reading titles and abstracts. Selected articles were then fully read to check their eligibility and quality; their references were reviewed for potential new studies to add. Disagreement among reviewers was resolved by discussion or consultation.

## 3. Results

### 3.1. Study Selection

**Figure 1:**
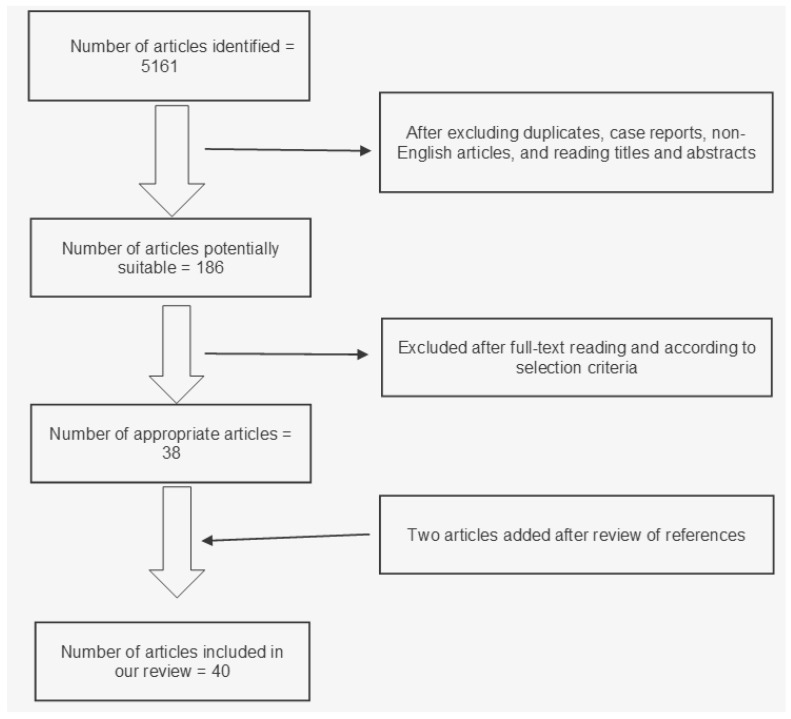
Flowchart of study selection for our review.

Our research strategy identified 5161 potentially relevant articles (Figure 1). After excluding duplicates, case reports, and non-English articles, we read titles and abstracts; after that, we still had 186 studies potentially suitable for our project. After full text reading and analysis, 38 articles were judged as “appropriate” for our review. Two articles were added after references were reviewed. In total, 40 articles were included in our review. More specifically, 8 of them had MF assessment as the main topic, 5 were studies about fibrosis quantification, and 27 were articles about fibrosis as a prognostic factor. Main characteristics of the included studies are shown in Table 1.

### 3.2. Fibrosis Assessment

About 10 years ago, the Magnetic Resonance Imaging (MRI) mapping sequences technique showed promising results in terms of MF assessment, for many cardiac diseases [8]. A CT scan-based fibrosis assessment, with good correlation with standard MRI, has been described as well [9]. Over the last few years, some authors have described a combined assessment using imaging (MRI) and laboratory markers (hs cTrI, NT-proBNP), showing good correlation [10]. Nowadays, fibrosis assessment may be accurately performed through imaging and biomarker studies, but we need to focus on fibrosis phenotyping and, subsequently, on specific anti-fibrosis targets to improve HF management and outcomes [11]. Combined assessment (MRI, echocardiography, NT-proBNP) also has been described for children with hypertrophic cardiomyopathy, showing interesting results [12].

Living myocardial slices are cultured and manipulated in vitro to investigate fibrosis biology, sometimes applying mechanical load to resemble physiological behavior [13,14]. MF being a heterogeneous and dynamic process, a reliable in vitro model is mandatory to better understand its pathophysiological and clinical aspects; novel techniques have been developed to move forward in this basic field [15].

### 3.3. Fibrosis Quantification

Cardiac MRI provides a simple means of detecting MF in a variety of pathologies; in addition to being non-invasive and carrying little risk (especially in patients with preserved kidney function), the whole heart can be examined within a short time, providing a more accurate diagnosis than localized, invasive procedures. Cardiac MRI has shown great results in measuring and quantifying fibrosis through Late Gadolinium Enhancement (LGE) and T1 mapping; also, the non-invasive nature of MRI allows for long-term tracking of fibrotic remodeling processes when serial measurements of MF are acquired [16]. MF quantification can be performed using both imaging and histology, with good correlation for ex vivo rat hearts [17]. However, the relationship between MRI findings and biomarkers is not totally understood yet [18]. Some studies have described the use of contrast-free MRI (T2 sequence) for mapping and fibrosis quantification in animal models, with good results [19]. Currently, assessment and quantification can be even performed for a specific heart chamber, as for atrial fibrosis in the settings of atrial fibrillation (AFib) [20].

### 3.4. Fibrosis as Prognostic Factor and Potential Target

Some authors have considered how MF may influence clinical outcomes in patients with HF [21]. Specific fibrosis phenotypes, assessed through endomyocardial biopsies, have been related to poor clinal outcomes [22]. Use of LGE was already described as a predictor of ventricular arrhythmias in patients with ventricular dysfunction [23]. LGE is a powerful and independent predictor of malignant arrhythmic prognosis, but its amount and distribution does not provide additional prognostic value [24]. Later, a meta-analysis showed a correlation between left ventricular mid-wall LGE and risk of all-cause mortality and cardiovascular mortality [25]. A recent systematic review described the high prognostic value of LGE in predicting poor outcomes in patients with ischemic cardiomyopathy (ICM); it may also provide helpful information for clinical decision-making related to sudden death prevention, and improve risk stratification, prognostication, and selection of patients with ICM for implantable cardioverter defibrillator (ICD) [26]. Detection of fibrosis through LGE MRI can be considered as a useful pathway of prediction of malignant arrhythmias since it has excellent prognostic characteristics and may help guide risk stratification and management in patients with non-ischemic dilated cardiomyopathy [27]. Correlation between fibrosis and mortality has been shown in patients after ICD implantation and, in contrast, mortality was not associated with LVEF; that suggested the use of fibrosis quantification rather than LVEF to select patients for ICD implantation [28,29]. Fibrosis progression was associated with increased hazards for all-cause mortality and heart failure-related complications [30]. MF was described as a predictor of sudden death in patients with coronary artery disease as well and a strong predictor of hospitalization for HF and death in patients with myocardial infarction [31,32]. In patients with primary mitral valve regurgitation, fibrosis was associated with mitral prolapse and with higher incidence of arrhythmic events [33]. Recently, an echocardiogram-based algorithm has been proposed to predict cardiovascular events and fibrosis in patients with mitral valve prolapse [34]. The REMODEL study showed that in patients with hypertension, MF is associated with adverse cardiac remodeling and outcomes like acute coronary syndrome, HF hospitalization, strokes, and cardiovascular mortality [35]. A meta-analysis showed that ischemic MF detected by LGE was associated with an increased risk of major cardiac and cerebrovascular events in patients with diabetes and may be an imaging biomarker for risk stratification [36]. Prognostic association of MF was demonstrated in patients with HIV [37]. Extracellular volume (ECV) measures of diffuse fibrosis were associated with HF outcomes despite exclusion of replacement fibrosis segments from their derivation and even among patients without any scar; moreover, ECV may have a synergistic role with LGE in HF risk assessment [38]. In patients with aortic regurgitation, indexed ECV was more strongly associated with AR severity and adverse clinical outcomes than ECV or replacement fibrosis [39]. The role of ECM could influence clinical outcomes: in an animal study, Metalloproteinase 9 knockout mice (which lost ECM regulation) had larger infarction size, lower ejection fraction, and higher mortality than wild-type mice [40]. However, results of studies about circulating levels of ECM biomarkers in relation to cardiac fibrosis and reverse cardiac remodeling have been conflicting, limited by small sample sizes, the variety of available commercial assays to measure biomarkers, the degree of correlation between circulating biomarker levels and the cardiac measure or mechanism that is being considered, and the molecular stability of biomarkers [41]. A treatment comparison between Levosimendan and Levosimendan plus ivabradine showed that the latter combination can effectively alleviate MF in patients [42].

N-cadherin is a calcium-dependent glycoprotein that mediates intercellular adhesion; its deletion resulted in dilated cardiomyopathy; slow conduction and higher conduction anisotropy and then ventricular tachycardia in a mouse model; loss of T-tubules (crucial for synchronization of myofilament contraction), suggesting that surface structure alterations are an integral part of the remodeling process during cardiac failure; and altered gap junction (due to down- and mis-regulation of channels) which leads to conduction block and reentrant arrhythmias [43]. Connexin-43 (Cx43) protein is a component of gap junctions in ventricular cardiomyocytes. Its migration from polar ends to lateral surface of the cell is linked to abnormal cardiomyocyte-to-myofibroblast coupling and the associated risk of arrhythmia; for this reason, Cx43 has been proposed as a potential target for prevention of aberrant cardiomyocyte coupling and myofibroblast proliferation in the infarct border zone [44]. Recently, authors showed that Beta-2 adrenergic receptors and L-type Ca^2+^ channels form a complex which is lost in heart failure [45]. Exosomes (small extracellular vesicles) also have a role in fibrosis development and the use of stem cell-derived and/or engineered exosomes as anti-fibrotic agents has been proposed [46]. In non-diabetic mellitus mice, Empagliflozin may regulate adenosine monophosphate-activated protein kinase mammalian target of rapamycin complex 1 pathway to attenuate adverse cardiac remodeling and progression of HF induced by pressure overload [47]. Sphingosine 1-phosphate receptor modulator fingolimod attenuates the collagen formation and hydroxyproline content after experimental transplantation, demonstrating a reduction in cardiac fibrosis [48].

**Table 1:** List and characteristics of included studies.

Author and Year	Title	Type of Study	Main Topic	Findings
Ambale-Venkatesh (2014) [16]	Cardiac MRI: a central prognostic tool in myocardial fibrosis	Systematic Review	Fibrosis as a prognostic factor	T1 mapping has shown significant potential for detecting diffuse MF, offering information on the ECV and providing valuable prognostic data for conditions like HCM, amyloidosis, sarcoidosis, and fibrosis associated with hypertension, diabetes, and aging.
Balycheva et al. (2015) [43]	Microdomain-specific localization of functional ion channels in cardiomyocytes: an emerging concept of local regulation and remodelling	Systematic Review	Fibrosis assessment	The functional alteration of protein–protein interactions and the disruption of normal subcellular targeting of ion channels and associated signaling proteins are linked to the development of MF and related cardiac conditions, including heart failure and arrhythmias. Disruptions in Cx43 phosphorylation are associated with fibrosis and arrhythmias, and lateralization of Cx43 appears in dilated and hypertrophic cardiomyopathies, correlating with arrhythmogenicity.
Beliveau et al. (2015) [17]	Quantitative Assessment of Myocardial Fibrosis in an Age-Related Rat Model by Ex Vivo Late Gadolinium Enhancement Magnetic Resonance Imaging with Histopathological Correlation	Laboratory study	Fibrosis quantification	LGE MRI and histology showed a good correlation in fibrosis quantification, with elderly rats having increased collagen content compared to young rats. Texture analysis also demonstrated a higher signal-to-noise ratio and a strong correlation with histology.
Black et al. (2024) [32]	Remote myocardial fibrosis predicts adverse outcome in patients with myocardial infarction on clinical cardiovascular magnetic resonance imaging	Clinical study	Fibrosis as a prognostic factor	Infarct size had a weak correlation with remote MF, showing that other factors contribute significantly to fibrosis development. Remote MF was also significantly associated with the risk of first hospitalization for heart failure and death. Infarct size and LVESVi lost significance after multivariable adjustment.
Centurión et al. (2019) [27]	Myocardial Fibrosis as a Pathway of Prediction of Ventricular Arrhythmias and Sudden Cardiac Death in Patients With Nonischemic Dilated Cardiomyopathy	Review	Fibrosis as a prognostic factor	Fibrosis, particularly in the mid-wall region, creates a substrate for ventricular arrhythmias, through slow and heterogeneous conduction, promoting reentry mechanisms. In LGE-positive patients, there is a risk for higher rates of arrhythmic events, making LGE a stronger predictor of arrhythmias than LVEF alone. LVEF and LGE detection by MRI may improve risk stratification for ICD therapy, allowing more accurate identification of patients at high risk of ventricular arrhythmias.
Chery et al. (2020) [26]	Prognostic value of myocardial fibrosis on cardiac magnetic resonance imaging in patients with ischemic cardiomyopathy: A systematic review	Systematic Review	Fibrosis as a prognostic factor	LGE has strong prognostic value in predicting adverse outcomes in patients with ICM. Increased MF burden was associated with a higher risk of arrhythmias and all-cause mortality. In spite of heterogeneity in scar parameters, LGE consistently correlated with adverse outcomes, demonstrating its role in risk stratification beyond conventional parameters like LVEF.
Disertori et al. (2016) [23]	Myocardial Fibrosis Assessment by LGE Is a Powerful Predictor of Ventricular Tachyarrhythmias in Ischemic and Nonischemic LV Dysfunction: A Meta-Analysis	Meta-analysis	Fibrosis as a prognostic factor	The composite arrhythmic endpoint occurred in 23.9% of patients with positive LGE versus 4.9% with negative LGE. LGE showed a strong correlation with arrhythmic events, with a pooled OR of 5.62 (95% CI: 4.20 to 7.51). In patients with LVEF ≤30%, the OR increased to 9.56, with a significant predictive value for arrhythmic events.
Disertori et al. (2017) [28]	Myocardial fibrosis predicts ventricular tachyarrhythmias	Review	Fibrosis as a prognostic factor	Fibrosis assessment: LGE is highlighted as a powerful non-invasive tool for detecting MF. Fibrosis quantification: presence and extent of MF were strong predictors of ventricular arrhythmias across several heart diseases. Prognostic factor: fibrosis, especially when detected by LGE, is shown to correlate significantly with ventricular arrhythmias and SCD, making it a valuable marker for risk stratification in patients with LV dysfunction.
Huttin et al. (2024) [34]	A new evidence-based echocardiographic approach to predict cardiovascular events and myocardial fibrosis in mitral valve prolapse: The STAMP algorithm	Clinical study	Fibrosis as a prognostic factor	Four distinct echocardiographic phenotypes were identified. Clusters with more pronounced remodeling had a higher prevalence of MF and were associated with increased risk of cardiovascular events. LV and LA remodeling and MF can occur independently of the severity of MR. STAMP algorithm, which incorporated echocardiographic variables such as MR severity, LV strain, and LA volume, successfully stratified patients into different risk categories.
Iyer et al. (2022) [35]	Markers of Focal and Diffuse Nonischemic Myocardial Fibrosis Are Associated With Adverse Cardiac Remodeling and Prognosis in Patients With Hypertension: The REMODEL Study	Clinical study	Fibrosis as a prognostic factor	Patients with nonischemic LGE had significantly greater LV mass, worse cardiac function, and higher levels of biomarkers for myocardial stress and injury. Both nonischemic LGE and indexed interstitial volume were indpendently associated with adverse outcomes, including heart failure hospitalization and cardiovascular mortality. Patients with the best prognosis were those without LGE and without a rise in interstitial volume.
Kholmovski et al. (2019) [20]	Cardiac MRI and Fibrosis Quantification	Review	Fibrosis quantification	Atrial fibrosis is a significant predictor of outcomes in AFib ablation and HF. LGE MRI is useful for quantifying fibrosis/scar in LA, which can predict ablation outcomes. Atrial fibrosis can be a modifiable risk factor for improving ablation procedures. Advanced atrial fibrosis is linked to worse long-term outcomes.
Kirmani et al. (2023) [12]	Cardiac imaging and biomarkers for assessing myocardial fibrosis in children with hypertrophic cardiomyopathy	Clinical study	Fibrosis assessment	Both echocardiography and cardiac MRI techniques showed good agreement for LV dimensions and septal thickness measurements, although MRI is more accurate, particularly when considering interventions like ICD placement. NT-proBNP levels were associated with LV mass and interventricular septal thickness, and cardiac troponin-T showed a marginal association with LV mass. The high prevalence of MF in children underscores the importance of early detection and monitoring.
Kitkungvan et al. (2018) [33]	Myocardial Fibrosis in Patients with Primary Mitral Regurgitation with and Without Prolapse	Clinical study	Fibrosis as a prognostic factor	Patients with MVP showed higher prevalence of LV replacement fibrosis compared to non-MVP patients. MVP patients also had greater LV mass, larger LV and RV volumes, larger left atrial volumes, and more severe MR, but LV systolic function did not differ significantly between the groups. MVP patients with LV replacement fibrosis had the highest rate of arrhythmic events, suggesting that replacement fibrosis in MVP patients may serve as an arrhythmic substrate, contributing to ventricular arrhythmias.
Langer et al. (2015) [9]	Myocardial Fibrosis in Hypertrophic Cardiomyopathy: Volumetric Assessment of Late Enhancement Provided by Cardiac Computed Tomography	Clinical study	Fibrosis assessment and quantification	leMDCT and LGE MRI were compared in 30 HCM patients. leMDCT detected late enhancement in 63.3% of cases, matching LGE MRI with 100% sensitivity. leMDCT had lower contrast-to-noise and signal-to-noise ratios compared to LGE MRI. Both methods showed high agreement in quantifying MF.
Lee et al. (2022) [19]	Quantification of Myocardial Fibrosis using Noninvasive T2-mapping Magnetic Resonance Imaging: Preclinical Models of Aging and Pressure Overload	Laboratory study	Fibrosis quantification	Statistical agreement between T2-map-quantified MF and Picro Sirius red staining ex vivo analysis in two different animal models. T2-mapping MRI is a promising non-invasive contrast-agent-free quantitative technique to characterize MF.
Leuw et al. (2021) [37]	Myocardial Fibrosis and Inflammation by CMR Predict Cardiovascular Outcome in People Living with HIV	Clinical study	Fibrosis as a prognostic factor	Participants with higher native T1 and T2 times, indicative of increased MF, had worse cardiovascular outcomes. Traditional risk scores like Framingham Risk Score were not predictive. Native T1 measurements and other MRI-based markers have the possibility to enhance cardiovascular risk assessment in populations with conditions that affect the heart differently than the general population, such as those living with HIV.
Leyva et al. (2022) [29]	Myocardial Fibrosis Predicts Ventricular Arrhythmias and Sudden Death After Cardiac Electronic Device Implantation	Clinical study	Fibrosis as a prognostic factor	Quantifying fibrosis significantly improved risk prediction. Higher GZF mass was strongly associated with both SCD and arrhythmic events. All patients who experienced SCD had MF with MFVA on preimplantation CMR, leading to 100% negative predictive value, meaning that absence of MFVA virtually ruled out the risk of SCD. LVEF did not predict SCD or arrhythmic events.
Li et al. (2021) [47]	Direct Cardiac Actions of the Sodium Glucose Co-Transporter 2 Inhibitor Empagliflozin Improve Myocardial Oxidative Phosphorylation and Attenuate Pressure-Overload Heart Failure	Laboratory study	Fibrosis as a prognostic factor	Empagliflozin significantly increased survival rates, reduced MF and myocardial hypertrophy, and improved both systolic and diastolic function in TAC-induced heart failure. It improved cardiomyocyte contractility and calcium handling and decreased glycolysis.
Li et al. (2021) [10]	Predictive values of multiple non-invasive markers for myocardial fibrosis in hypertrophic cardiomyopathy patients with preserved ejection fraction	Clinical study	Fibrosis quantification and as a prognostic factor	LGE-positive patients showed significantly higher levels of Nt-proBNP hs-cTnI compared to LGE-negative patients. Nt-proBNP ≥ 108.00 pg/mL and MWT ≥ 17.30 mm had good diagnostic accuracy for identifying fibrosis, with a sensitivity of 70.00% and a specificity of 81.25%. GCS was more sensitive than GLS in detecting MF in HCM patients.
Lin et al. (2022) [42]	Diagnostic value of cardiac magnetic resonance imaging for myocardial fibrosis in patients with heart failure and its predictive value for prognosis	Clinical study	Fibrosis as a prognostic factor	The group treated with levosimendan combined with ivabradine hydrochloride demonstrated better improvement in cardiac function, lower levels of MF markers (ICTP, PIIINP, CTGF, HA, LN), and better physical recovery and quality of life compared to the group treated with levosimendan alone. LGE was effective in identifying patients with MF, and the study found that higher levels of LVESV, LVESD, and lower LVEF could predict fibrosis.
Mandawat et al. (2021) [30]	Progression of Myocardial Fibrosis in Nonischemic DCM and Association with Mortality and Heart Failure Outcomes	Clinical study	Fibrosis as a prognostic factor	Fibrosis remained stable in 82% of patients, but in 18% of patients showed progression and was associated with worsening LV remodeling, decreased LVEF, and higher risks of all-cause mortality and heart failure complications. When fibrosis progressed, many patients exhibited minimal changes in LVEF, highlighting fibrosis progression as an independent marker of risk.
Marra et al. (2014) [24]	Impact of the presence and amount of myocardial fibrosis by cardiac magnetic resonance on arrhythmic outcome and sudden cardiac death in nonischemic dilated cardiomyopathy	Clinical study	Fibrosis as a prognostic factor	MF was detected using LGE MRI, and 55.5% of patients were found to have LV-LGE. The presence of LV-LGE was identified as a strong and independent predictor of malignant arrhythmias and SCD. However, the extent and distribution of LGE did not add additional prognostic value. The findings suggest that LV-LGE detected by MRI can be used to better identify patients at high risk for SCD, even when they do not meet traditional criteria (LVEF) for ICD implantation.
Nunez-Toldra et al. (2022) [14]	Mechanosensitive molecular mechanisms of myocardial fibrosis in living myocardial slices	Laboratory study	Fibrosis assessment and quantification	LMS under mechanical overload showed an increase in fibrosis-related markers and reduced contractility. IL-11 enhanced fibrosis and reduced contractility, mimicking the effects of mechanical overload. Treatment with a TGF-βR blocker reduced fibrotic remodeling under mechanical overload and improved contractility. The model creates a physiologically relevant 3D environment, offering insights into the mechanosensitive molecular mechanisms driving fibrosis and serving as a model for testing anti-fibrotic therapies.
O’Meara et al. (2023) [41]	Fibrosis Biomarkers Predict Cardiac Reverse Remodeling	Clinical study	Fibrosis as a prognostic factor	Lower baseline levels of PICP were associated with improvements in LV reverse remodeling and better clinical outcomes, such as reduced cardiovascular mortality. Patients with lower PICP levels and LVEF at one year showed the best prognosis. High PICP levels were associated with worse outcomes, regardless of LVEF improvement.
Perbellini et al. (2018) [13]	Investigation of cardiac fibroblasts using myocardial slices	Laboratory study	Fibrosis assessment	Myocardial slices maintain physiological interactions of CF, succeeding in avoiding culture-induced phenotypic changes such as over-expression of α- typical of myofibroblasts. Application of mechanical load significantly reduces CF proliferation and helps maintain the structural and functional integrity of the slices.Pharmacological stimulation with TGF-β and ANG II failed to induce CF activation as seen in traditional cell culture models and cells do not express α-SMA.
Pichler et al. (2020) [18]	Cardiac magnetic resonance-derived fibrosis, strain and molecular biomarkers of fibrosis in hypertensive heart disease	Clinical study	Fibrosis assessment and fibrosis as a prognostic factor	ECV and strain were found to correlate with changes in cardiac geometry and function, indicating early MF and dysfunction. ECV was significantly associated with LA diameter and longitudinal strain, while biomarkers like CITP were only marginally related to strain. Molecular biomarkers, like collagen degradation markers, showed a weak relationship with MRI-derived fibrosis, indicating that MRI might be a more reliable tool.
Pitoulis et al. (2022) [15]	Remodelling of adult cardiac tissue subjected to physiological and pathological mechanical load in vitro	Laboratory study	Fibrosis assessment	Both pressure- and volume-overloaded LMS showed hypertrophic remodeling with increased cardiomyocyte size. Pressure-overloaded LMS exhibited concentric remodeling, while volume-overloaded LMS showed eccentric remodeling. Unique pathways demonstrated in volume overload included cardiac muscle development and sarcomeric organization, whereas pressure-overloaded LMS showed enrichment in pathways related to inflammation, stress response, and metabolism.
Ravassa et al. (2023) [11]	Cardiac Fibrosis in heart failure: Focus on non-invasive diagnosis and emerging therapeutic strategies	Systematic Review	Fibrosis assessment and quantification	Biomarkers like miR-21, TGF-β, and PICP show correlations with myocardial collagen deposition in heart failure; however, many biomarkers are not specific to the heart. MRI is the most effective non-invasive imaging tool for visualizing focal and diffuse fibrosis. LGE and T1 mapping with ECV quantification are key for detecting and monitoring fibrosis. ACE inhibitors and ARBs have shown histological evidence of reducing MF and improving LV function.
Ravassa et al. (2017) [22]	Phenotyping of myocardial fibrosis in hypertensive patients with heart failure. Influence on clinical outcome	Clinical study	Fibrosis quantification and as a prognostic factor	Low serum levels of CITP and high serum PICP can identify patients with excessive collagen cross-linking and collagen deposition, characterizing them as having a high-risk MF phenotype. These patients exhibited a higher probability of heart failure hospitalization and cardiovascular or all-cause death over a 5-year period.
Roller et al. (2015) [8]	T1, T2 Mapping and Extracellular Volume Fraction (ECV): Application, Value and Further Perspectives in Myocardial Inflammation and Cardiomyopathies	Systematic Review	Fibrosis quantification	Elevated native T1 values in fibrosis-affected areas, compared to healthy myocardium, were observed in various conditions: HCM, DCM, and AFD. Post-contrast T1 mapping helps detect diffuse fibrosis by quantifying changes in myocardial relaxation times, with lower post-contrast T1 times indicating fibrotic areas. Higher ECV values are indicative of more extensive fibrosis and show higher precision compared to traditional T1 mapping techniques. ECV is particularly effective in differentiating between healthy myocardium and areas affected by diffuse fibrosis, providing a non-invasive method to assess the severity of fibrosis.
Sanchez-Alonso et al. (2023) [45]	Functional LTCC-β2AR Complex Needs Caveolin-3 and Is Disrupted in Heart Failure	Laboratory study	Fibrosis assessment	In cardiomyocytes from failing hearts, the β2AR-LTCC coupling is impaired, with reduced LTCC response. PKA inhibition partially preserved β2AR effects, while CaMKII inhibition blocked them. Disruption of caveolae led to a loss of β2AR-LTCC coupling. In human cardiomyocyte data, β2AR stimulation enhances LTCC activity in healthy but not in CMD samples, indicating preserved coupling in normal conditions and its disruptionin disease.
Schelbert et al. (2019) [21]	Myocardial Scar and Fibrosis The Ultimate Mediator of Outcomes?	Review	Fibrosis as a prognostic factor	Excess in interstitial protein deposition, like in amyloidosis, leads to adverse outcomes in both conditions, including heart failure, microvascular dysfunction, and ventricular arrhythmia. RAAS-targeting medications show modest success in improving heart function, but have not significantly reversed MF and have been less effective in patients with heart failure with preserved ejection fraction.
Schultz et al. (2019) [44]	Cardiomyocyte–myofibroblast contact dynamism is modulated by connexin-43	Laboratory study	Fibrosis assessment	Silencing Cx43 reduced dynamism in CM–MFB and MFB–MFB contacts, emphasizing the importance of functional gap junctions. The molecule 4PB increased coupling and reduced dynamism in CM–MFB pairs and hypoxia decreased dynamism due to Cx43 internalization. The aCT1 peptide increased CM-MFB dynamism, and actin filament inhibition with latrunculin-B decreased dynamism, highlighting the role of actin in Cx43-mediated interactions.
Senapati et al. (2021) [39]	Regional Replacement and Diffuse Interstitial Fibrosis in Aortic Regurgitation: Prognostic Implications From Cardiac Magnetic Resonance	Clinical study	Fibrosis as a prognostic factor	iECV and iCV significantly increased with AR severity, but ECV and replacement fibrosis did not change significantly with AR severity. Only LVEF was associated withreplacement fibrosis. iECV, which reflects total LV fibrosis burden, was associated with AR severity and adverse clinical outcomes and was a better predictor of clinical outcomes than ECV. In patients with increasing AR severity, both iECV and iCV increased, reflecting extracellular matrix and cellular hypertrophy, while ECV remained unchanged due to balanced cellular and extracellular expansion.
Sung et al. (2020) [40]	Losing Regulation of the Extracellular Matrix is Strongly Predictive of Unfavorable Prognostic Outcome after Acute Myocardial Infarction	Laboratory study	Fibrosis as prognostic factor	The mortality rate after AMI was significantly higher in DKO mice compared to wild-type mice. DKO mice exhibited larger infarct areas, reduced LVEF, and increased fibrosis and collagen depositionin myocardium. The biomarkers of apoptosis, fibrosis, oxidative stress, and inflammation were significantly elevated in DKO mice and angiogenesis markers were reduced in DKO mice.
Tikhomirov et al. (2020) [46]	Exosomes: From Potential Culprits to New Therapeutic Promise in the Setting of Cardiac Fibrosis	Review	Fibrosis assessment	Exosomes play a key role in intercellular communication and can carry pro-fibrotic or anti-fibrotic signals, depending on their content. Exosomes derived from stem cells or engineered exosomes show promise as therapeutic agents in reducing fibrosis and improving cardiac function. There are a few challenges in exosome-based therapy: variability in exosome composition and the need for improved isolation techniques.
Wang et al. (2020) [25]	Left ventricular midwall fibrosis as a predictor of sudden cardiac death in non-ischaemic dilated cardiomyopathy: a meta-analysis	Meta-analysis	Fibrosis as a prognostic factor	LV midwall LGE was present in 30.8% of NICM patients and was associated with an increased risk of all-cause mortality (OR 3.37) and cardiovascular mortality (OR 5.56). SCD or aborted SCD events (OR 2.25). LV midwall LGE pattern has a high negative predictive value, which means that NICM patients with this pattern are at high risk and may benefit from ICD therapy regardless of LVEF.
Yang et al. (2022) [36]	Association of myocardial fibrosis detected by late gadolinium-enhanced MRI with clinical outcomes in patients with diabetes: a systematic review and meta-analysis	Meta-analysis	Fibrosis as a prognostic factor	MF detected by LGE MRI significantly increased the risk of MACCEs and MACEs. The presence of ischemic fibrosis was especially associated with these outcomes, suggesting that LGE MRI could be used as a prognostic biomarker in diabetic patients. Even if the risk for MACCEs and MACEs was significantly increased, LVEF persisted.
Yang et al. (2019) [38]	Myocardial Extracellular Volume Fraction Adds Prognostic Information Beyond Myocardial Replacement Fibrosis	Clinical study	Fibrosis quantification	Higher ECV, independent of myocardial scar, was significantly associated with heart failure hospitalization and mortality and also provided additional prognostic information beyond LGE and other traditional risk factors, suggesting its value as a risk marker for HF outcomes.
Zegard et al. (2021) [31]	Myocardial Fibrosis as a Predictor of Sudden Death in Patients with Coronary Artery Disease	Clinical study	Fibrosis as a prognostic factor	Patients with MF and GZF mass greater than 5.0 g had a significantly higher risk of SCD and arrhythmic events than those without fibrosis. GZF mass was a stronger predictor than LVEF, which is traditionally used for risk stratification; this suggests that quantifying GZF mass may be preferable for deciding on the need for implantable ICDs in CAD patients.

MF—Myocardial Fibrosis; ECV—Extracellular Volume; HCM—Hypertrophic Cardiomyopathy; MRI—Magnetic Resonance Imaging; LGE—Late Gadolinium Enhancement; LVESVi—Left Ventricular End Systolic Volume Index; LVEF—Left Ventricular Ejection Fraction; ICD—Implantable Cardioverter–Defibrillator; ICM—Ischemic Cardiomyopathy; OR—Odds Ratio; SCD—Sudden Cardiac Death; LV—Left Ventricle; LA—Left Atrium; MR—Mitral Regurgitation; AFib—Atrial Fibrillation; HF—Heart Failure; NT-proBNP—N-terminal pro-brain natriuretic peptide; MVP—Mitral Regurgitation with Prolapse; leMDCT—Late-enhanced Multislice Computed Tomography; GZF—Gray Zone Fibrosis; MFVA—Myocardial Fibrosis Present on Visual Assessment; TAC—Transverse Aortic Constriction; hs-cTnI—High-Sensitivity Cardiac Troponin I; GLS—Global Longitudinal Strain; GCS—Global Circumferential Strain; AMI—Acute Myocardial Infarction; CTGF—Connective Tissue Growth Factor; ICTP—Carboxy-terminal Telopeptide of Type I Collagen; PIIINP—Amino-terminal Propeptide of Type III Procollagen; PICP—Procollagen Type 1 Carboxy-terminal Peptide; HA—Hyaluronic Acid; LN—Laminin; LVESV—Left Ventricular End Systolic Volume; LVESD—Left Ventricular End Systolic Diameter; LMS—Living Myocardial Slices; CF—Cardiac Fibroblasts; DCM—Dilated Cardiomyopathy; AFD—Anderson–Fabry Disease; PKA—Protein Kinase A; CaMKII—Calcium–Calmodulin-Dependent Protein Kinase II; LTCC—L-type Ca^2+^ Channels; RAAS—Renin–Angiotensin–Aldosterone System; CM—Cardiomyocytes; MFB—Myofibroblasts; iECV—Indexed Extracellular Volume; iCV—Indexed Collagen Volume; AR—Aortic Regurgitation; DKO—Double Knockout Mouse; NICM—Non-Ischemic Cardiomyopathy; MACCE—Major Adverse Cardiovascular and Cerebrovascular Events; MACE—Major Adverse Cardiac Events; CAD—Coronary Artery Disease.

## 4. Discussion

MF is a crucial process involved in most cardiovascular diseases. Over the last few years, we have taken important steps forward regarding both pathophysiology and clinical features, but some aspects are yet to be explained. We aim to present the current state-of-the-art about MF.

Assessment of MF is a primary step in our clinical practice. In the past, diagnosis of MF was strictly histologic; nowadays, it may be performed through imaging (usually MRI) having accurate information [8,10,11]. Among imaging tools, cardiac MRI showed the best results in terms of assessment and it currently is safely used both for diagnostic assessment and follow-up. After MF assessment became a reliable diagnostic tool, MF quantification was the next natural step. MRI was demonstrated to be the best imaging instrument to do that; specific T1 and T2 mapping sequences are used to obtain detailed information [16,19]. Mapping is useful to show where and how much MF is present in a heart, showing excellent correlation with histologic quantification [17]. Having these findings, we may argue that, in the mid-term, MRI could also substitute for myocardial biopsies, being less invasive and less risky for the patient; moreover, it gives information about the entire heart, not just about a small specimen as a biopsy does. LGE extent is another way to quantify MF, giving us defined information about scarring by probing the retention of contrast agent. Contrast-free MRI is a new described method to quantify MF [19]. Gadolinium toxicity, despite being rare, has been described especially in patients with chronic kidney disease [49]. For this reason, the use of a contrast-free imaging exam would be very helpful to reduce complications in that cohort of patients. This could be a promising, less-invasive tool for the near future. Another important move is the chamber- and/or disease-specific MF quantification, as in the settings of AFib [20]. Afib is a common disease and MF is a key factor in determining both pathophysiological background and clinical aspects. Mapping and quantification of MF could be pivotal in the future to improve percutaneous treatments of this disease.

When quantification was becoming more accurate, authors wondered about how MF may influence clinical outcomes in patients with HF. Many authors described the relationship between MF and arrhythmias [23,24,26,28]. Some proposed MF as a prognostic factor —rather than LVEF—and as an indication parameter for ICD implantation [28,29]. Currently LVEF less than 30–35% is classically the indication to implant an ICD [50]. In the near future, we could use a specific MF pattern (or a MF quantity cut-off) as an indication for ICD, covering patients with better LVEF who have high arrhythmic risk as well, who currently have no indication for ICD (hypertrophic and restrictive cardiomyopathies).

We now know that MF is not a static, amorphic entity. Both active fibroblasts (myofibroblasts) and ECM communicate with myocytes and several abnormal changes have been described in pathologic myocardium; Balycheva and Schultz described the mechanisms which lead to altered gap junctions and abnormal myocyte–fibroblast coupling, having as a result an arrhythmogenic pattern [43,44]. This is relevant in order to find a possible, specific feature which may be related to poor outcomes or a potential therapeutical target. We may speculate that, although fibrosis pathophysiology is not totally understood yet, MF is involved in several chronic heart diseases, and in the future it could be used as a prognostic factor in many scenarios other than HF or arrhythmias. To do that, however, we will need to enhance the basic and translational science.

## 5. Future Directions

In the future, the role of MF in clinical practice will be more significant than now. Assessment techniques will be more accurate and less invasive: the next-generation, 7-tesla MRI will give us extremely detailed information. Moreover, MF quantification could become a determining point in patients with HF to evaluate the need for ICD implantation, likely replacing the role of LVEF. Mapping probably will be more used to plan percutaneous (and maybe surgical) treatment of AFib. In terms of assessment and quantification, MRI could also substitute myocardial biopsies. As previously stated, MRI is safer and less invasive, and it gives information about the heart in toto. Basic science will play a key role in improving our knowledge about MF: the use of dynamic, living myocardial slices and reliable in vitro models are unavoidable to make progress. The final goal is to find a specific anti-fibrosis target to use in patients with cardiac diseases to slow down or even reverse the process which leads to HF and malignant arrhythmias.

## 6. Conclusions

MF is a crucial process in patients with cardiac diseases and leads to HF and arrhythmias. Non-invasive assessment and quantification have taken great steps forward in recent years and are now used routinely, but more research is needed to strengthen the role of MF as a prognostic factor in the future. Basic science will play a key role in finding specific anti-fibrosis treatments.

## Data Availability

No new data were created or analyzed in this study. Data sharing is not applicable.
